# The Third Study of Infectious Intestinal Disease (IID3 Study) in the Community: Protocol for UK-Based Prospective Cohort Studies Investigating the Disease Burden

**DOI:** 10.2196/88759

**Published:** 2026-02-25

**Authors:** Ben W Rowland, Vanashree Sexton, Aileen Mill, Stephen Rushton, Roy Sanderson, Caroline Grundy, Simon de Lusignan, Nigel A Cunliffe, Daniel Hungerford, Mark Hopkins, Saheer Gharbia, Claire Jenkins, Gauri Godbole, Roberto Vivancos, Alex J Elliot, Dominic J Mellor, Lesley Larkin, Rachel Chalmers, Sarah O'Brien, IID3 Consortium

**Affiliations:** 1 School of Natural and Environmental Sciences Newcastle University Newcastle upon Tyne, England United Kingdom; 2 Clinical Informatics and Health Outcomes Research Group Nuffield Department of Primary Care Health Science University of Oxford Oxford, England United Kingdom; 3 Department of Clinical Infection, Microbiology and Immunology Institute of Infection, Veterinary and Ecological Sciences University of Liverpool Liverpool, England United Kingdom; 4 Liverpool Clinical Laboratories Liverpool University Hospitals NHS Trust Liverpool, England United Kingdom; 5 Wellcome Sanger Institute Hinxton, England United Kingdom; 6 UK Health Security Agency London, England United Kingdom; 7 Emerging and Zoonotic Infections National Institute for Health Research Liverpool, England United Kingdom; 8 Warwick Medical School University of Warwick Coventry, England United Kingdom; 9 National Institute for Health Research University of East Anglia Norwich United Kingdom; 10 NIHR Health Protection Research Unit in Emergency Preparedness and Response University of Birmingham Birmingham United Kingdom; 11 Public Health Scotland Edinburgh, Scotland United Kingdom; 12 Swansea University Swansea, Wales United Kingdom; 13 See Acknowledgements

**Keywords:** infectious intestinal disease, IID, cohort, general practitioner, sample, pathogen

## Abstract

**Background:**

There is a significant hidden burden of infectious intestinal disease (IID) in the UK community, which has increased over time. In the late 2000s, the Second Study of Infectious Intestinal Disease (IID2 study) estimated 17 million IID cases annually in the United Kingdom. However, only a small proportion of cases present to health care, and even those are often not tested for causative organisms.

**Objective:**

The Third Study of Infectious Intestinal Disease (IID3 study) aims to determine the IID burden in the UK community, estimate the underreporting level in routine practice and the general population, and recalibrate UK national surveillance based on the new incidence rates.

**Methods:**

We will follow methods of previous studies, along with modern pathogen detection methods and digital platforms for recruitment and follow-up. Participants will be recruited to three population-based prospective cohorts: cohort 1 (the general population), cohort 2 (patients with IID presenting to general practices [GPs]), and cohort 3 (enumeration study of IID cases presenting to GPs). Microbiological analysis of stool samples in cohorts 1 and 2 will include testing for a wide range of causative organisms using molecular assays, including pathogen targets not routinely sought by National Health Service (NHS) laboratories. Additional characterization of pathogens will be conducted at national reference laboratories. The incidence rates of IID and organisms detected in cohorts 1-3 will be compared to national surveillance systems, both laboratory and syndromic. Descriptive statistics and analysis will allow comparison of IID rates within each cohort, estimate the overall burden of disease caused by different pathogens, and compare findings to earlier IID studies.

**Results:**

A favorable ethical opinion was obtained from the UK Health Research Authority on August 4, 2022. A pilot phase to test the sampling process was conducted from January to August 2023. Participant recruitment commenced on September 1, 2023, for cohort 2 and on March 16, 2024, for cohort 1; recruitment ceased on August 31, 2025. Data collection is complete, and data analysis is to begin. The study is expected to end in September 2026.

**Conclusions:**

Since the first and second IID studies, changes have occurred within national surveillance systems, the NHS structure, and public recommendations about when to consult a GP and where to seek health care advice, which may have altered the extent of IID reporting and the perceived burden in the community, creating greater uncertainty about the representativeness of IID rates. The IID3 study results will provide insight into trends in disease incidence over time and help quantify inequalities in IID in the UK community. Revised estimates can inform policy related to prevention, including food standards and disease management. Furthermore, advances in molecular diagnostics will significantly enhance pathogen detection, increasing our understanding of the causes of IID.

**International Registered Report Identifier (IRRID):**

DERR1-10.2196/88759

## Introduction

### Background

Infectious intestinal disease (IID) is a common illness, with an estimated 17 million cases in the United Kingdom each year [1]. In 2018, the estimated cost of foodborne illness to the UK economy was approximately GBP 9 (USD 12.4) billion per year [2]. Although most cases are self-limiting, these infections can lead to significant complications and mortality, with substantial age and pathogen-specific inequalities [3,4]. IID is caused by a wide range of causative organisms, including bacteria (eg, *Salmonella* spp. and *Campylobacter* spp.), viruses (eg, norovirus and rotavirus), and parasites (eg, *Giardia* spp. and *Cryptosporidium* spp.). Transmission of these pathogens occurs through a variety of routes, including foodborne, waterborne, and person-to-person transmission, as well as contaminated environments [5-7]. Foodborne transmission alone accounts for an estimated 2.4 million cases in the United Kingdom annually. Only a small proportion of people with IID report to primary care, but their illness may prevent them from attending work or school. In the Second Study of Infectious Intestinal Disease (IID2 study; 2007-2009) [1], a major, unexpected result was that this hidden burden of disease had increased between the First Study of Infectious Intestinal Disease (IID1 study; 1993-1996) [8] and the IID2 study, largely due to changes in the delivery of primary care. The societal and economic impact of IID and the resulting health inequalities were greatly underestimated because they could not be measured through routine surveillance, which relies on contact with health services.

Understanding the level of underreporting is important because public health actions by regulators, such as the Food Standards Agency (FSA), need to be based on robust information that accurately identifies hazards and risks. Much of the current information used by policy advisors is based on the results of IID2. However, uncertainty is growing about the reliability of IID2 results to accurately reflect the current status of IID rates and reporting practices in the United Kingdom, as older estimates inevitably become less accurate over time.

Furthermore, four major changes make recalibrating national surveillance data particularly important to the FSA’s core work. First, significant changes to primary care delivery are still evolving. Following the COVID-19 pandemic, there has been an increase in digital and non–general practice (GP)–based access to primary care and advice (eg, triage via National Health Service [NHS] 111/NHS 24 and pharmacies) [9]. These changes likely result in fewer cases of disease being directly reported, and in the absence of independent estimates to support revised underreporting figures, the allocation of funding and investment in prevention and disease management is not well informed.

Second, the public health and social measures enacted during the COVID-19 pandemic had a substantial impact on the confirmed cases and reported outbreaks of IID (34% and 52% decrease, respectively) [10]. This was reflected in national surveillance data, with the rates of disease being reported having returned to prepandemic levels [11]. Despite this, it is important to consider the impacts of the COVID-19 pandemic on current reporting patterns of disease, engagement in research, and how and when people access the health care system for routine care.

Third, laboratory diagnostic methods have changed significantly over time. Improved molecular methods have expanded the range and sensitivity of pathogen detection, enabling a more complete description of infections and potentially altering previous estimates. For example, in the IID1 study, traditional bacterial culture methods were used, whereas in the IID2 study, a mix of traditional and molecular methods (polymerase chain reaction [PCR]) were used. The Third Study of Infectious Intestinal Disease (IID3 study) will use molecular methods as the primary diagnostic approach for detecting bacteria, viruses, and parasites, reflecting the shift by NHS laboratories toward molecular methods for pathogen detection.

Fourth, infant immunization against rotavirus IID was introduced in the United Kingdom in 2013, and vaccine uptake rapidly exceeded 90%. Before vaccine introduction, rotavirus was estimated to account for 80,000 GP consultations and 750,000 episodes of diarrhea [12,13]. Since the introduction, there have been substantial reductions in health care–attended rotavirus-associated gastroenteritis among vaccine-eligible children [14,15]. There is also some evidence of indirect effects in older unvaccinated populations. Therefore, the burden and epidemiology of rotavirus-associated IID is likely to be substantially different since the IID2 study was conducted. It is, therefore, timely to conduct the third UK study of IID to quantify the impact of changes in the epidemiology of infection and disease reporting, and to investigate established and emerging pathogens. The design of the IID3 study will allow for the identification of underreporting rates across primary care to assess whether the societal burden of IID has changed since the late 2000s. There is an opportunity to pivot over time toward lower-cost, continuous sentinel surveillance, but these approaches need to be benchmarked against conventional ones.

### Study Overview

The IID3 study comprises three population-based prospective studies (one cohort study and two studies in primary care), supported by diagnostic and reference microbiological investigation of fecal specimens. Routine public health surveillance data, provided by the United Kingdom Health Security Agency (UKHSA), Public Health Scotland (PHS), Public Health Wales (PHW), and the Public Health Agency Northern Ireland (PHA NI), allow calibration of the IID incidence rates reported to national surveillance records.

### Overall Study Aims

The IID3 study aims to determine the incidence rates of and identify the microorganisms associated with IID in the community, as well as incidence rates of IID presenting to GPs; compare these data with routine testing in primary care in the United Kingdom; and investigate how this has changed since the previous IID2. The seven study aims are as follows:

- Determine the overall incidence of IID in the UK population.
- Establish the incidence of IID presenting to primary care.
- Clarify the proportion of IID that is UK acquired.
- Describe the pathogens causing IID in the United Kingdom, including levels of antimicrobial resistance (AMR).
- Recalibrate UK surveillance data for IID, overall and by pathogen.
- Determine the number of cases in the community, GP-reported cases, and hospitalizations and deaths due to IID in the United Kingdom.
- Compare results from the IID3 study to previous studies.

## Methods

### Study Setting and Case Definition

Participants and GPs were recruited from across all four UK nations. We used the same case definition used for the earlier IID studies to ensure direct comparability [16]. Thus, “cases” were defined as “persons with loose stools or clinically significant vomiting lasting less than 2 weeks, in the absence of a known noninfectious cause, preceded by a symptom-free period of 3 weeks.” Vomiting is considered clinically significant if it occurs more than once in a 24-hour period and if it incapacitates the case or is accompanied by other symptoms, such as cramps or fever.

### Study Design

**Figure 1:**
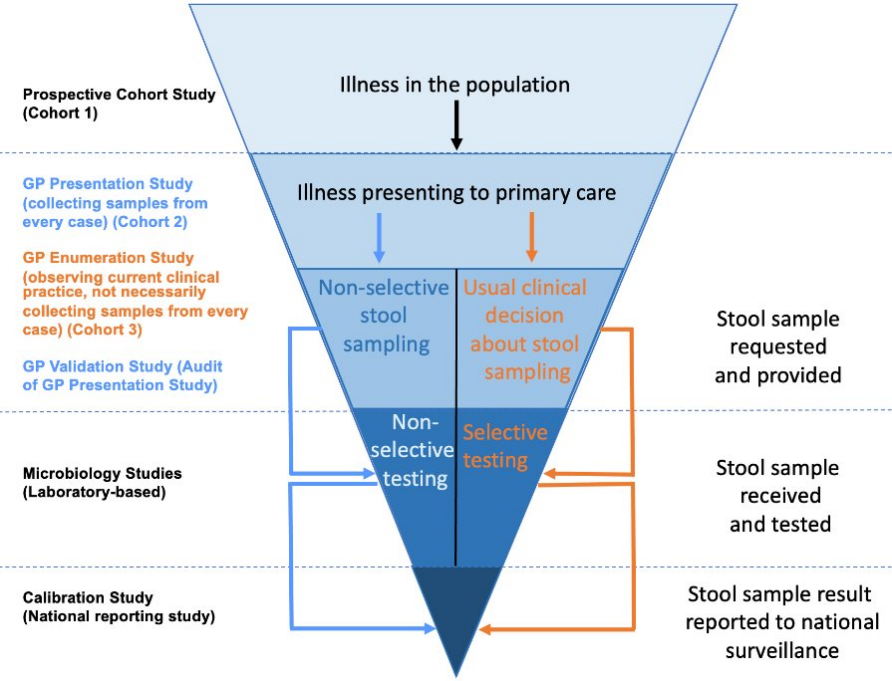
Overview of study design. GP: general practice.

The IID3 study comprises three prospective cohorts: cohort 1 (prospective population), cohort 2 (GP presentation), and cohort 3 (GP enumeration). The three cohorts are described in detail later and in Figure 1, which shows the study’s reverse pyramid structure. Recruitment into the cohorts and record-linked access to primary care routine data from GPs were enabled through the Oxford Royal College of General Practitioners (RCGP) Research and Surveillance Centre (RSC) [17].

#### Prospective Cohort Study (Cohort 1)

Cohort 1 is a prospective, population-based household group designed to identify the incidence of IID in the community who may not present to GPs. In addition, cohort 1 data will provide insights into the transmission of IID within households. Participants with no current symptoms of IID were recruited to this cohort via invitations to join the study from their GPs. The invitations were sent in batches via text messages to the majority of the GP-registered population using clinical management software to apply the study’s exclusion criteria (see Textbox 1).

**Patient eligibility criteria.**

**Inclusion criteria:**

- Patients with diarrhea or clinically significant vomiting lasting less than 2 weeks, with no known noninfectious cause, preceded by a symptom-free period of 3 weeks
- Any age

**Exclusion criteria (used for cohort 1 participant recruitment):**

- Patients with terminal illness
- Patients with severe mental incapacity
- Patients who do not speak English and for whom a suitable interpreter is not available
- Surgical obstruction

Participants’ IID status (whether they had experienced any symptoms of vomiting and/or diarrhea) was followed up weekly for 12 months through the study mobile app (PeopleWith). If participants reported symptoms, they were asked to complete a symptom questionnaire (see the full questionnaire in Multimedia Appendices 1 and 2) and to submit a stool sample.

#### GP Presentation Study (Cohort 2)

Cohort 2 is a prospective cohort of patients presenting to primary care with symptoms of IID. GPs were instructed to invite all patients who presented with IID symptoms (as per the case definition). Recruitment was primarily opportunistic, with patients presenting with IID symptoms asked to participate in the study following a face-to-face or telephone consultation. Patients who consented were asked to complete a symptom questionnaire (see the full questionnaire in Multimedia Appendix 3). GPs participating in cohort 2 collected stool samples from recruited patients.

#### GP Enumeration Study (Cohort 3)

The enumeration study will provide contemporary data on routine practice through a 24-month prospective audit of IID cases presenting to GPs, observing them as they occur in routine clinical practice. We included all RSC practices, excluding participating in cohorts 1 and 2 to prevent any participant from appearing in two cohorts, and compiled data on the clinical severity of IID cases, stool sample submission, and microbiology laboratory results. Systematized Nomenclature of Medicine Clinical Terms (SNOMED CT) codes will be used to identify IID cases.

#### Validation Study

To assess the level of underascertainment of IID cases in cohort 2 (where patients meeting the case definition were not invited or did not consent to the study), the participating practices will be audited by identifying all IID cases using the same SNOMED CT codes as the enumeration study.

#### Calibration Study

The overall IID incidence rates and pathogen-specific incidence rates will be calculated for each cohort. Cohort 1 data will provide a rate for the general population; cohort 2 data will allow us to estimate the true level of IID illness among those seeking primary care; and cohort 3 data will provide a rate observed in routine primary care. Comparison between these cohorts and with rates of IID reported to national surveillance systems will allow estimation of the degree of underreporting to primary care and national surveillance systems.

### Data Sources

#### Participant and Symptom Information

Participant consent, baseline information, and information about IID symptoms were collected for cohorts 1 and 2 through a bespoke mobile app. The app was linked to a web-based portal that allows GPs to view participants who have provided consent, which was monitored by the study team to track recruitment. For cohort 1, GPs used the portal to view when participants reported illness on their weekly symptom questionnaires and to send an automated email asking participants to collect a stool sample kit.

Participants in cohort 1 who reported IID symptoms or those in cohort 2 who presented to GPs with IID were asked to complete the symptom questionnaire to obtain data on the following items: symptoms experienced and number of days the symptoms lasted; date of symptom onset; prior health care service use for symptoms, including use of the NHS 111 telephone service or other urgent care services; and recent travel outside the United Kingdom. The complete questionnaire is available in Multimedia Appendices 1 and 2.

The RSC provided primary care medical record data for cohorts 1, 2, and 3. These data include participant-associated variables, such as age, ethnicity, index of multiple deprivation (IMD), records of vaccination history, recent changes to medication, antibiotic usage (within the 3 months prior to the start of the study), referrals to gastrointestinal (GI) specialists, a history of GI or respiratory illness, and details on pregnancy.

### Stool Microbiology

Participants in cohorts 1 and 2 who reported symptoms of IID were requested to supply a stool sample. Samples were subject to PCR testing at the Liverpool Clinical Laboratories, Liverpool University Hospitals NHS Foundation Trust, to identify the presence of causative organisms (see Table 1 for a list of organisms sought). Commercial assays used for this purpose included Serosep EntericBio DX, Viral and *Clostridioides difficile* assays detecting 15 pathogens, plus additional PCR tests (Genetic Signatures) that target *Clostridium perfringens, Aeromonas* spp., *Cyclospora cayetanensis*, and diarrhoeagenic *Escherichia coli* (enterotoxigenic, enteropathogenic, and enteroaggregative strains) [18]. Routine diagnostic laboratory results from cohorts 1 and 2 were reported to the participants’ general practitioners.

**Table 1:** Causative organisms tested in stool samples using PCR^a^.

Bacterium		Virus	Parasite
*Clostridioides difficile*		Adenovirus	*Cryptosporidium parvum/hominis*
*Clostridium perfringens*		Astrovirus	*Giardia lamblia*
*Campylobacter jejuni/coli/lari*		Norovirus	*Cyclospora cayetanensis*
**Diarrheagenic** ***Escherichia coli*** **(DEC)**		Rotavirus A	*Entamoeba histolytica*
	Enteroaggregative *E. coli* (DEC_EAEC)	Sapovirus	—^b^
	Enteropathogenic *E. coli* (DEC_EPEC)	—	—
	Enterotoxigenic *E. coli* (DEC_ETEC)	—	—
*Salmonella enterica* spp.		—	—
*Shigella* spp.		—	—
Shiga toxin-producing *Escherichia coli* (STEC)		—	—
*Yersinia enterocolitica* spp.		—	—
*Aeromonas* spp.		—	—
*Vibrio cholerae/parahaemolyticus*		—	—

^a^PCR: polymerase chain reaction.

^b^Not applicable.

Further characterization of selected pathogens identified using these primary diagnostic methods will be carried out at the UKHSA’s Gastrointestinal Bacteria Reference Unit (bacteria), Enteric Virus Unit (viruses), and Public Health Wales *Cryptosporidium* Reference Unit (parasites).

Cohort 3 data will enable us to report on routine clinical practice, where any stool samples will follow standard diagnostic routes; no additional testing will be performed for these cases. We received data on typing, bacterial multilocus sequence typing (MLST), multiple-locus variable number (MVLA) for parasites, and single-nucleotide polymorphism (SNP) addresses, as well as a variety of AMR data.

#### Sample Archiving

Stool samples from participants who provided consent for storage are archived at the University of Liverpool’s Good Clinical Practice (GCP) Laboratory Biobank for potential use in future research. A sample aliquot is saved in Shield Fluid (Zymo Research) to preserve nucleic acids for future molecular applications, where the material allows.

### National Surveillance

#### Laboratory Pathogen Surveillance

National surveillance data on GI pathogens were provided for each of the four UK nations by the UKHSA, the PHS, the PHW, and the PHA NI. To ensure standardization from these sources, we developed a common script to be used across the four nations to extract and deduplicate the data. Preprocessing and checking of these datasets ensured deduplication based on patient name, NHS number, and date, as well as pathogen genus, species, and serotype. The data provided vary depending on availability by country, but broadly, the datasets include International Organization for Standardization (ISO) week and year, 5-year age group (<1 year, 1-4 years, 5-9 years, and so on), sex, postcode district, whether the case was imported (the UKHSA only), measure of deprivation (eg, the English IMD, Scottish IMD, and Welsh IMD), organism (genus, species, type, and subtype), and antimicrobial sensitivity to different antimicrobials where such tests have been performed.

#### Syndromic Surveillance

Syndromic surveillance data, in the form of nonidentifiable records of phone calls relating to IID made to the national telephone health services NHS 111 (England) and NHS 24 (Scotland), were also provided by the UKHSA and the PHS, respectively. These data include date of call, syndrome (symptoms reported by the caller, limited to diarrhea or vomiting), 5-year age group, sex, and postcode district.

### Data Storage and Security

Data collected for the study were pseudonymized and stored within a secure, trusted research environment (TRE) operated by the University of Oxford. Where available, cohort 1 and cohort 2 sample collection was facilitated through the Lab Links system. The system was intended to minimize the study-related workload of sample processing by enabling practices to follow their usual clinical sample workflow for study samples, allowing them to order tests and receive results. However, as this was not available to most practices, an alternative manual approach, where sample kits were to be returned by post, was provided to participating GPs and patients, as required.

### Data Linkage

For cohorts 1 and 2, data from three sources can be linked to an individual participant: the participant’s information and symptom data from the study mobile app, results from the study laboratory, and medical history information from the primary care record. The datasets are linked within the secure Oxford Royal College of General Practitioners, Clinical Informatics Digital Hub (ORCHID) TRE using an encrypted key that is consistent across the three data sources. Cohort 3 data will be cross-checked to ensure no study participant (cohort 1 and cohort 2 participants) appears in the cohort 3 dataset.

### Ethical Considerations

Ethical opinion was sought from the UK Health Research Authority using the Integrated Research Applications System (IRAS). Further information governance approvals were obtained from the Public Benefit and Privacy Panel for Health and Social Care (NHS Scotland). A consortia-wide study agreement outlines partner roles and responsibilities, and data-sharing agreements were put in place between organizations for data or material transfer. Approval for the study was obtained from the RCGP, which is a policy-level data controller of the RSC. As mentioned earlier, data were pseudonymized and stored within the secure ORCHID TRE. Access to the data is limited to data analysts on the IID3 study team. The ORCHID TRE meets the requirements of NHS Digital’s Data Security and Protection (DSP) Toolkit and is compliant with university policies and relevant legislation. Informed consent was obtained from all adults before entry to the study. For children, consent was sought from a parent or guardian.

### Data Analysis

#### Assessing Representativeness

Representativeness and inequalities will be assessed by comparing the characteristics of the study population with those from routine data from the national census and GP statistics. We will quantify inter-GP variation in patient characteristics, including sociodemographic features associated with the patient population, using mixed-effect modeling. Seasonal variation in the data will be removed through detrending, and factors driving residual nonseasonal disease variation will be determined using count negative binomial (generalized) linear mixed-effects models. Detrending will occur by including harmonic variables (sine and cosine of time) as covariates in the linear mixed-effect models [19]. Model fit will be assessed by comparing Akaike information criterion (AIC) or similar values between models, comparing R^2^ values, and examining model diagnostics, such as QQ plots. If any remaining temporal autocorrelation remains in the data, it will be adjusted for using autoregression.

#### Assessing Compliance

Compliance in cohorts 1 and 2 will be calculated as the proportion of participants submitting a questionnaire or stool sample relative to the total number of participants who had confirmed participation in the presentation study. Differences in compliance across age, sex, or practice groups/areas will be assessed using mixed-effects analyses with GP as a random effect; a Pearson chi-square test and multiple logistic regression will be used.

#### Assessing Underascertainment

Underascertainment in cohorts 1 and 2 will be investigated by comparing the frequency of IID diagnosis in the GP presentation study data with that ascertained in the enumeration study. This will also be used to assess the likely contribution of unmeasured GP variation on ascertainment. Factors influencing underascertainment will be investigated using multiple logistic regression in a mixed-effects framework (ie, generalized linear mixed-effects models with a binomial error structure). The inverse of the predicted underascertainment from this model will be used as a multiplier for the number of cases reported in each practice, considering practice characteristics and intraclass correlation from the mixed-effects models.

### Estimating the IID Incidence

We will exploit the hierarchical structure of patients within a GP as our modeling framework. All analyses of the incidence of individual IID pathogens will be based on PCR results; bacterial PCR-positive samples will be subject to routine culture and identification to allow comparison with the IID1 and IID2 studies.

#### Incidence Rate of IID

The incidence rate of IID in the community will be calculated using the total number of cases reported in the population (cohort 1) and the follow-up time of cohort members. Follow-up periods will be censored for times when participants were not considered at risk (ie, while traveling). Incidence rates of IID will be calculated as an overall rate and adjusted to account for age and sex.

#### Incidence Rate of IID Cases Presenting to the GP

The incidence rate of IID cases presenting to GPs (cohort 2) will be calculated using the total number of cases reported in the presentation and enumeration components (cohorts 2 and 3, respectively) and the follow-up time of all cohort members registered in the practice populations. The numerator and denominator of the GP presentation rates will be adjusted to account for underascertainment and list inflation. Information about individual patient symptoms will be incorporated into the analyses, as appropriate.

### The Role of Individual Pathogens

#### Number of Cases by Organism

For cohorts 1 and 2, age and seasonality incidence will be estimated for each target organism using denominators derived from the age/sex registers of the study practices and the sampled population. Organism-specific incidence rates will be calculated from the number of cases in which each target organism was identified, regardless of whether other organisms were present in the same sample. Underascertainment will be assumed to be the same for all organisms. Rates will be adjusted for noncompliance with submitting stool samples based on overall compliance data. We will assume that noncompliant cases have the same identification rate for each organism as compliant cases. Calculation of CIs will be adjusted to ensure that there is no increase in the apparent precision due to this correction for noncompliance*.*

### Comparing the Etiology of IID and Surveillance Pyramids

#### Incidence Rates and Presentation Rates

Incidence rates and presentation rates (overall for IID and by target organism) will be compared between the IID1 and IID2 studies and the new IID3 study based on the incidence in this research estimated using the laboratory methods used in the first study; changes in incidence in the community and presentation to GPs will be estimated, together with their associated 95% CIs calculated using a Wald test.

#### Surveillance Pyramids

Surveillance pyramids will be created using the overall community incidence rates and presented to GPs, with ratios between these rates and their respective CIs included. The ratio of community rates to nationally reported cases will be calculated by projecting the overall cohort and presentation incidence rates to the population and comparing these to ratios reported to the laboratory reporting surveillance system over the same period. When more than one laboratory method is used, the results from both methods will be estimated separately for comparison with the IID1 study and as a baseline for the future.

#### Three Key Components of the Surveillance Pyramids

Three key components of the surveillance pyramids will be compared across the IID1 and IID2 studies and the new IID3 study: overall incidence rates in the community, presentation to GPs, and reporting to the laboratory surveillance system. A simple comparison will be made between these ratios. Performing the same calculation on the upper and lower CIs for each ratio will derive the upper and lower sensitivity bands.

#### Molecular Data

Molecular data used to derive some of the surveillance pyramids in the IID1 and IID2 studies were obtained using different molecular methods than those available for the IID3 study. The surveillance pyramid for the IID3 study can be recalculated assuming that the molecular techniques previously used with different targets are now in place. This will allow a more direct comparison between the new IID3 surveillance and the original IID1 and IID2.

### Patient and Public Involvement and Engagement

We will have public and patient involvement and engagement (PPIE) at all stages of the research. We received input from patient participation groups in primary care, the PPIE research theme and panel from the NIHR Health Protection Research Unit in Gastrointestinal Infections, and PPIE representatives on the IID3 Study Executive Committee and the External Advisory Panel. We sought and incorporated feedback on how to phrase questions about sex/gender and the material’s reading level for children. A primary school teacher gave us feedback, and they said the material should be suitable for a reading age of 7-8 years. We also incorporated feedback from older members of the community to ensure the material is unlikely to cause offence. PPIE involvement will continue throughout the study’s life cycle.

## Results

A favorable ethical opinion was obtained from the UK Health Research Authority on August 4, 2022 (IRAS ID 314268, REC Reference 22/EM/0130). A pilot phase to test the sampling process was conducted from January to August 2023. Participant recruitment commenced on September 1, 2023, for cohort 2 and on March 16, 2024, for cohort 1; participation in both cohorts ceased on August 31, 2025 [20]. Data collection is complete, and data analysis is to begin. The results will be disseminated to key stakeholders, including the FSA, participating GPs, and the academic community, through reports, publications, and conference presentations. The study is expected to end in September 2026. The study-generated data will be archived after study completion and will be available to the general public.

## Discussion

### Summary

Our ability to determine the true burden of IID in a population remains a challenge, as only a small proportion of cases appear in national surveillance statistics. Large-scale interdisciplinary prospective cohort studies are critical to maintaining our understanding of disease incidence and causative pathogens. This study is timely as it will provide new estimates of the disease burden postpandemic and also follows the introduction of the new rotavirus vaccine in 2013 in children under 5 years of age. New pathogen-specific incidence rates will inform policy and pathogen management, such as the rollout of the norovirus vaccine currently under development.

All stool samples in the study were subject to sensitive molecular assays, using significant advancements in molecular diagnostics since the IID2 study, to allow detection of a broad range of bacteria, viruses, and parasites. Further characterization of positive samples by national reference laboratories in England and Wales will provide additional information about the strain types circulating in the community. The enhanced microbiological testing will also improve our understanding of pathogen detection through routine surveillance and where this could be improved. The revised incidence rates across the study surveillance pyramids will allow recalibration of the national surveillance of IID, while accounting for improvements in our ability to detect disease since the previous estimates.

The IID3 study methodology will provide valuable insight into changing patterns of primary care reporting practices following the worldwide COVID-19 pandemic. The past 5 years have seen substantial changes to how patients access primary care in the United Kingdom, with more people relying on electronic communications (NHS 111/NHS 24 and GP messaging systems) to report illness and seek advice, when they may previously have had an appointment with their GP. We anticipate this to be reflected by a change in IID incidence rates for the GP presentation cohort.

### Strengths and Limitations

Although the study uses the same overall methodology as the previous IID1 and IID2, the digital recruitment approach for cohort 1 is novel, using a direct message system to reach the community population through GP registrations [20]. Participant follow-up was conducted via a mobile app to enable real-time collection of weekly questionnaire data, removing the need for paper-based communications and data entry.

We recognize that digital study recruitment may have limited access to the study to those who are permanently registered with a GP and have access to a phone or email to complete the weekly questionnaires. Recruitment of children (under 18 years) was challenging due to limitations with direct contact and consenting minors through text message invitation and the mobile app, which may result in underrepresentation of viral causes of IID, as these are likely to be more dominant in young children. However, embedding the study within the Oxford RCGP Research and Surveillance Centre primary care sentinel surveillance network has facilitated recruitment from nationally representative research-ready GPs with experience in undertaking primary care research, including on IID [17].

### Conclusion

Here, we describe the protocol of the IID3 study in the United Kingdom, which will provide contemporary estimates of the disease burden in the UK population. Updated IID reporting and incidence rates are urgently needed to reflect changes in primary care interaction and improved diagnostic capability and to recalibrate national surveillance reporting. This prospective cohort study approach will provide comparable rate estimates to previous studies (IID1 and IID2 studies), putting us in the unique position of being able to report trends over three decades. The World Health Organization, national governments, and public health organizations rely on these robust estimates of disease incidence and underreporting as evidence to inform policy agendas, interventions. and decision-making.

## Data Availability

The data collected from the Third Study of Infectious Intestinal Disease (IID3 study) will be archived in the UK Data Archive at the end of the study (September 2026). At this time, the data will be publicly available. National surveillance data will not be archived and will require data-sharing agreements with the relevant public health agency to obtain.

## Appendix

Multimedia Appendix 1 Cohort symptom questionnaire for adults.

Multimedia Appendix 2 Cohort symptom questionnaire for children.

Multimedia Appendix 3 GP symptom questionnaire. GP: general practice.

## Abbreviations

AMR: antimicrobial resistance
FSA: Food Standards Agency
GI: gastrointestinal
GP: general practice
IID: infectious intestinal disease
IID1 study: First Study of Infectious Intestinal Disease
IID2 study: Second Study of Infectious Intestinal Disease
IID3 study: Third Study of Infectious Intestinal Disease
IMD: index of multiple deprivation
NHS: National Health Service
ORCHID: Oxford Royal College of General Practitioners, Clinical Informatics Digital Hub
PCR: polymerase chain reaction
PHA NI: Public Health Agency Northern Ireland
PHS: Public Health Scotland
PHW: Public Health Wales
PPIE: public and patient involvement and engagement
RCGP: Royal College of General Practitioners
RSC: Research and Surveillance Centre
SNOMED CT: Systematized Nomenclature of Medicine Clinical Terms
TRE: trusted research environment
UKHSA: United Kingdom Health Security Agency
