# Supplementary material for: The Third Study of Infectious Intestinal Disease (IID3 Study) in the Community: Protocol for UK-Based Prospective Cohort Studies Investigating the Disease Burden
Source: JMIR Res Protoc. 2026 Feb 25;15:e88759. doi: 10.2196/88759 (PMC12980067; doi:10.2196/88759)
Supplement: Multimedia Appendix 1 [file resprot_v15i1e88759_app1.doc]

**Symptom Questionnaire (Weekly Follow-up Study)**


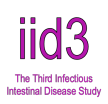


**The Third Study of Diarrhoea and Vomiting in the Community**

**IRAS ID: 314268** ***For Official Use Only***

Participant’s Study Number:

We want to know how often people in the UK suffer from diarrhoea or vomiting and the germs that cause this. Thank you for agreeing to fill in this questionnaire.

***Please read each question carefully before you answer it, and try to answer every question. Please either tick the appropriate box or write your answer in the space provided.***

***The information that you give us will be treated in strict confidence.***

**Part 1: This section asks about your age and sex**

Please tell us:

1.1 Today’s date (dd/mm/yyyy): / /

1.2 Your date of birth (dd/mm/yyyy): / /

1.3 Your sex: Male Female

Prefer not to say Prefer to self-identify

**Part 2: This section asks about the symptoms you had during your recent illness**

- 1. Did you have any of the following symptoms? For EACH symptom please tick Yes, No or Not sure.

**Diarrhoea**: (loose watery bowel movements)

Yes No Not sure

Number of days:

Still Present: Yes No Not sure

**Diarrhoea** **with blood in it:**

Yes No Not sure

Number of days:

Still Present: Yes No Not sure

**PLEASE TURN OVER**

**Nausea (feeling sick):**

Yes No Not sure

Number of days:

Still Present: Yes No Not sure

**Vomiting (being sick):**

Yes No Not sure

Number of days:

Still Present: Yes No Not sure

**Abdominal cramps (colic):**

Yes No Not sure

**Loss of appetite:**

Yes No Not sure

**Fever (high temperature):**

Yes No Not sure

**Cough or runny/blocked nose or sore throat:**

Yes No Not sure

**Headache:**

Yes No Not sure

2.2 What was the date (dd/mm/yyyy) on which you first had diarrhoea and/or vomiting?

/ /

2.3 If you answered “yes” to having diarrhoea, roughly how many times did you go to the toilet on the worst day (24 hours) of your illness?

Number of times

2.4 If you answered “yes” to vomiting, roughly how many times did you vomit on the worst day (24 hours) of your illness?

Number of times

**PLEASE TURN OVER**

2.5 Have you phoned NHS111 about this illness?

Yes No Not sure

If “yes”, on what date (dd/mm/yyyy) did you first phone NHS

111 about these symptoms?

/ /

2.6 Have you contacted the out-of-hours doctor service about this illness?

Yes No Not sure

If “yes”, on what date (dd/mm/yyyy) did you first contact the out-of-hours doctor service about these symptoms?

/ /

2.7 Have you visited a Walk-in centre about this illness?

Yes No Not sure

If “yes”, on what date (dd/mm/yyyy) did you first contact the walk-in-centre about these symptoms?

/ /

2.8 Have you spoken to your nurse or doctor on the ‘phone for advice about this illness?

Yes No Not sure

If “yes”, on what date (dd/mm/yyyy) did you first phone for advice about these symptoms?

/ /

2.9 Have you been to see a doctor or nurse in your practice about this illness?

Yes No Not sure

If “yes”, on what date (dd/mm/yyyy) did you first see your doctor

about these symptoms?

/ /

2.10 Did you go to hospital, Accident and Emergency (A&E) or casualty with this illness?

Yes No Not sure

If “yes”, on what date (dd/mm/yyyy) did you go to hospital, Accident and Emergency (A&E) or casualty about these symptoms?

/ /

**PLEASE TURN OVER**

2.11 Were you admitted to hospital overnight or longer with this illness?

Yes No Not sure

If “yes”, on what date (dd/mm/yyyy) were you admitted to hospital with this illness?

/ /

If “yes”, how many nights did you spend in hospital with this illness?

2.12 Did your illness stop you from going to work or to school or carrying out your daily activities?

Yes No Not sure

If “yes”, how many days?

**Part 3: This section asks about your travel in the ten days before you became ill.**

- 1. Did you travel outside the UK in the ten days before you became
     ill?

Yes No Not sure

3.2 If you answered “yes”, what dates (dd/mm/yy) were you away?

From: / / To: / /

3.3 If you were abroad, please tell us which country or countries you visited:

**Have you sent a faeces (stool) specimen?**

Yes No

If no, please do so as soon as possible, as this is really important for the study.

You can get another specimen pot from your practice if you do not have one.

**Thank you for taking the time to fill in this questionnaire.**
