# Supplementary material for: The Third Study of Infectious Intestinal Disease (IID3 Study) in the Community: Protocol for UK-Based Prospective Cohort Studies Investigating the Disease Burden
Source: JMIR Res Protoc. 2026 Feb 25;15:e88759. doi: 10.2196/88759 (PMC12980067; doi:10.2196/88759)
Supplement: Multimedia Appendix 2 [file resprot_v15i1e88759_app2.doc]

**Symptom Questionnaire (Weekly Follow-up Study)**


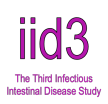


**The Third Study of Diarrhoea and Vomiting in the Community**

**The “Tummy Bug” Study**

**IRAS ID: 314268 *For Official Use Only***

Participant’s Study Number:

We want to know how often people in the UK suffer from diarrhoea or vomiting and the germs that cause this.

Thank you for agreeing to fill in this questionnaire.

***Please read each question carefully before you answer it, and try to answer every question. Please either tick the appropriate box or write your answer in the space provided.***

***The information that you give us will be treated in strict confidence.***

**Part 1: This section asks about your child’s age and sex**

Please tell us:

1.1 Today’s date (dd/mm/yyyy): / /

1.2 Child’s date of birth (dd/mm/yyyy): / /

1.3 Child’s sex: Male Female

**Part 2: This section asks about the symptoms your child had during your recent illness**

- 1. Did they have any of the following symptoms? For EACH symptom please tick Yes, No or Not sure.

**Diarrhoea**: (loose watery bowel movements)

Yes No Not sure

Number of days:

Still Present: Yes No Not sure

**Diarrhoea** **with blood in it:**

Yes No Not sure

Number of days:

Still Present: Yes No Not sure

**PLEASE TURN OVER**

**Nausea (feeling sick):**

Yes No Not sure

Number of days:

Still Present: Yes No Not sure

**Vomiting (being sick):**

Yes No Not sure

Number of days:

Still Present: Yes No Not sure

**Abdominal cramps (colic):**

Yes No Not sure

**Loss of appetite:**

Yes No Not sure

**Fever (high temperature):**

Yes No Not sure

**Cough or runny/blocked nose or sore throat:**

Yes No Not sure

**Headache:**

Yes No Not sure

2.2 What was the date (dd/mm/yyyy) on which your child first had diarrhoea and/or vomiting?

/ /

2.3 If you answered “yes” to having diarrhoea, roughly how many times did your child go to the toilet on the worst day (24 hours) of their illness?

Number of times

2.4 If you answered “yes” to having vomiting, roughly how many times did your child go to the toilet on the worst day (24 hours) of their illness?

Number of times

**PLEASE TURN OVER**

2.5 Have you phoned NHS111 about your child’s illness?

Yes No Not sure

If “yes”, on what date (dd/mm/yyyy) did you first phone NHS

111 about your child’s symptoms?

/ /

2.6 Have you contacted the out-of-hours doctor service about this illness?

Yes No Not sure

If “yes”, on what date (dd/mm/yyyy) did you first contact the out-of-hours doctor service about your child’s symptoms?

/ /

2.7 Have you visited a Walk-in centre about your child’s illness?

Yes No Not sure

If “yes”, on what date (dd/mm/yyyy) did you first contact the walk-in-centre about your child’s symptoms?

/ /

2.8 Have you spoken to your child’s nurse or doctor on the ‘phone for advice about their illness?

Yes No Not sure

If “yes”, on what date (dd/mm/yyyy) did you first phone for advice about these symptoms?

/ /

2.9 Have you been to your child’s doctor or nurse in your practice about this illness?

Yes No Not sure

If “yes”, on what date (dd/mm/yyyy) did your child first see their doctor

about these symptoms?

/ /

2.10 Did you take your child to hospital, Accident and Emergency (A&E) or casualty with this illness?

Yes No Not sure

If “yes”, on what date (dd/mm/yyyy) did you go to hospital, Accident and Emergency (A&E) or casualty about these symptoms?

/ /

**PLEASE TURN OVER**

- 1. Was your child admitted to hospital overnight or longer with this illness?

Yes No Not sure

If “yes”, on what date (dd/mm/yyyy) was your child admitted to hospital with this illness? / /

If “yes”, how many nights did your child spend in hospital with this illness?

2.12 Did your child’s illness stop them from going to school or day care?

Yes No Not sure

If “yes”, how many days?

**Part 3: This section asks about your travel in the ten days before your child became ill.**

- 1. Did your child travel outside the UK in the ten days before they became
     ill?

Yes No Not sure

3.2 If you answered “yes”, what dates (dd/mm/yy) were you away?

From: / / To: / /

3.3 If you were abroad, please tell us which country or countries your child visited:

**Have you sent a faeces (stool) specimen?**

Yes No

If no, please do so as soon as possible, as this is really important for the study.

You can get a specimen pot from your practice nurse.

**Thank you for taking the time to fill in this questionnaire.**
