# Supplementary material for: The Third Study of Infectious Intestinal Disease (IID3 Study) in the Community: Protocol for UK-Based Prospective Cohort Studies Investigating the Disease Burden
Source: JMIR Res Protoc. 2026 Feb 25;15:e88759. doi: 10.2196/88759 (PMC12980067; doi:10.2196/88759)
Supplement: Multimedia Appendix 3 [file resprot_v15i1e88759_app3.doc]

**Questionnaire (GP Presentation Study)**


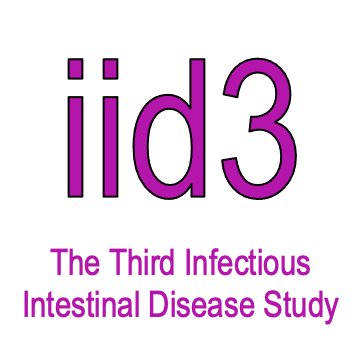


**The Third Study of Diarrhoea and Vomiting in the Community**

***For office use only***

Participant’s study number:

Date of consultation that led to study entry:

We want to know how often people in the UK suffer from diarrhoea or vomiting and the germs that cause this.

***Please read each question carefully before you answer it, and try to answer each question. Please either tick the appropriate box or write your answer in the space provided.***

***The information that you give us will be treated in strict confidence.***

**Part 1: This section asks for some background information about you.**

1. What is your surname: ………………………………………….

forename(s): ………………………………………….

2. What is your date of birth (dd/mm/yyyy)? ____/____/____­­­­­­­­­­­­­­­­­­­

3. Are you? Male Female

Prefer not to say Prefer to self-identify

4. Please give your address: ……………………………………………….….

……………………………………………………

……………………………………………………

5. What is your postcode?

6. What is your email address? ………………………………………………

7. How many people in total live in your household?

8. How many children live in your household?

9. How many bedrooms does your home have?

10. How many bathrooms does your household have?

Please tick one box. box.

11. Which ethnic group do you belong to?

| **White** | British or Irish |  |
| --- | --- | --- |
|  | Other |  |
| **Mixed** | White & Black Caribbean |  |
|  | White and Black African |  |
|  | White and Asian |  |
|  | Other Mixed |  |
| **Asian or Asian British** | Indian |  |
|  | Pakistani |  |
|  | Bangladeshi |  |
|  | Other Asian |  |
| **Black or Black British** | Black Caribbean |  |
|  | Black African |  |
|  | Other Black |  |
| **Another Group** | Chinese |  |
|  | Other ethnic group |  |

**PLEASE TURN OVER**

12. Please tick one box to show which **best** describes the sort of work the

**main earner** in your household does. (If the main earner is not working

now, please tick a box to show what they did in their last job).

Please tick one box.

| Modern professional occupations *such as:* teacher - nurse - physiotherapist - social worker - welfare officer - artist - musician - police officer (sergeant or above) - software designer |  |
| --- | --- |
| Clerical and intermediate occupations *such as:* secretary - personal assistant - clerical worker - office clerk - call centre agent - nursing auxiliary - nursery nurse |  |
| Senior managers or administrators (usually responsible for planning, organising and co-ordinating work and for finance)  *such as:* finance manager - chief executive |  |
| Technical and craft occupations *such as:* motor mechanic - fitter - inspector - plumber - printer -  tool maker - electrician - gardener - train driver |  |
| Semi-routine manual and service occupations *such as:* postal worker - machine operative - security guard - caretaker - farm worker - catering assistant - receptionist - sales assistant |  |
| Routine manual and service occupations *such as****:*** HGV driver - van driver - cleaner - porter - packer - sewing machinist - messenger - labourer - waiter / waitress - bar staff |  |
| Middle or junior managers *such as:* office manager - retail manager - bank manager, restaurant manager - warehouse manager - publican |  |
| Traditional professional occupations *such as:* accountant - solicitor - medical practitioner - scientist -  civil / mechanical engineer |  |

**PLEASE TURN OVER**

13. Last week, was the **main earner** in your home any of the following?

Please tick one box.

| Retired |  |
| --- | --- |
| Student |  |
| Looking after home/family |  |
| Currently sick/disabled |  |
| None of the above |  |

14. Does (did) the **main earner** work as an employee or are (were) they self-

Please tick one box.

employed?

| Employee |  |
| --- | --- |
| Self-employed with employees |  |
| Self-employed/freelance without employees  (please skip questions 12 and 13) |  |

15. **For employees**: indicate below how many people work (worked) for the

**main earner’s** employer at the place where they work (worked).

**For self-employed**: indicate below how many people the main earner

Please tick one box.

employs (employed).

| 1 to 24 |  |
| --- | --- |
| 25 or more |  |

16. Does (did) the **main earner** supervise any other employees?

A supervisor or foreman is responsible for overseeing the work of other

Please tick one box.

employees on a day-to-day basis

| Yes |  |
| --- | --- |
| No |  |

17. We may need to contact you at some time during the study.

What is the best telephone number to contact you on?

..........................................................

**PLEASE TURN OVER**

**Part 2: This section asks about the symptoms you had during your recent illness**

- 1. Did you have any of the following symptoms? For EACH symptom please tick Yes, No or Not sure.

**Diarrhoea**: (loose watery bowel movements)

Yes No Not sure

Number of days:

Still Present: Yes No Not sure

**Diarrhoea** **with blood in it:**

Yes No Not sure

Number of days:

Still Present: Yes No Not sure

**Nausea (feeling sick):**

Yes No Not sure

Number of days:

Still Present: Yes No Not sure

**Vomiting (being sick):**

Yes No Not sure

Number of days:

Still Present: Yes No Not sure

**Abdominal cramps (colic):**

Yes No Not sure

**Loss of appetite:**

Yes No Not sure

**PLEASE TURN OVER**

**Fever (high temperature):**

Yes No Not sure

**Cough or runny/blocked nose or sore throat:**

Yes No Not sure

**Headache:** Yes No Not sure

2.2 What was the date (dd/mm/yyyy) on which you first had diarrhoea and/or vomiting?

/ /

2.3 If you answered “yes” to having diarrhoea, roughly how many times did you go to the toilet on the worst day (24 hours) of your illness?

Number of times

2.4 If you answered “yes” to vomiting, roughly how many times did you vomit on the worst day (24 hours) of your illness?

Number of times

2.5 Have you phoned NHS111about this illness?

Yes No Not sure

If “yes”, on what date (dd/mm/yyyy) did you first phone NHS

111 about these symptoms?

/ /

2.6 Have you contacted the out-of-hours doctor service about this illness?

Yes No Not sure

If “yes”, on what date (dd/mm/yyyy) did you first contact the out-of-hours doctor service about these symptoms?

/ /

2.7 Have you visited a Walk-in centre about this illness?

Yes No Not sure

If “yes”, on what date (dd/mm/yyyy) did you first contact the walk-in-centre about these symptoms?

/ /

**PLEASE TURN OVER**

2.8 Have you spoken to your nurse or doctor on the ‘phone for advice about this illness?

Yes No Not sure

If “yes”, on what date (dd/mm/yyyy) did you first phone for advice about these symptoms?

/ /

2.9 Have you been to see a doctor or nurse in your practice about this illness?

Yes No Not sure

If “yes”, on what date (dd/mm/yyyy) did you first see your doctor

about these symptoms?

/ /

2.10 Did you go to hospital, Accident and Emergency (A&E) or casualty with this illness?

Yes No Not sure

If “yes”, on what date (dd/mm/yyyy) did you go to hospital, Accident and Emergency (A&E) or casualty about these symptoms?

/ /

2.11 Were you admitted to hospital overnight or longer with this illness?

Yes No Not sure

If “yes”, on what date (dd/mm/yyyy) were you admitted to hospital with this illness?

/ /

If “yes”, how many nights did you spend in hospital with this illness?

- 1. Did your illness stop you from going to work or to school or carrying out your daily activities?

Yes No Not sure

If “yes”, how many days?

**PLEASE TURN OVER**

**Part 3: This section asks about your travel in the ten days before you became ill.**

- 1. Did you travel outside the UK in the ten days before you became
     ill?

Yes No Not sure

3.2 If you answered “yes”, what dates (dd/mm/yy) were you away?

From: / / To: / /

3.3 If you were abroad, please tell us which country or countries you visited:

**Have you sent a faeces (stool) specimen?**

Yes No

If no, please do so as soon as possible, as this is really important for the study.

You can get another specimen pot from your practice nurse if you do not have one.

**Thank you for taking the time to fill in this questionnaire.**
